# A NDIR Mid-Infrared Methane Sensor with a Compact Pentahedron Gas-Cell

**DOI:** 10.3390/s20195461

**Published:** 2020-09-23

**Authors:** Weilin Ye, Zihan Tu, Xupeng Xiao, Alessandro Simeone, Jingwen Yan, Tao Wu, Fupei Wu, Chuantao Zheng, Frank K. Tittel

**Affiliations:** 1 Key Laboratory of Intelligent Manufacturing Technology, Ministry of Education, College of Engineering, Shantou University, 243 Daxue Road, Shantou 515063, China; wlye@stu.edu.cn (W.Y.); 18zhtu@stu.edu.cn (Z.T.); 19xpxiao@stu.edu.cn (X.X.); simeone@stu.edu.cn (A.S.); jwyan@stu.edu.cn (J.Y.); taowu@stu.edu.cn (T.W.); 2 State Key Laboratory of Integrated Optoelectronics, College of Electronic Science and Engineering, Jilin University, 2699 Qianjin Street, Changchun 130012, China; 3 Department of Electrical and Computer Engineering, Rice University, 6100 Main Street, Houston, TX 77005, USA; fkt@rice.edu

**Keywords:** infrared spectroscopy, mid-infrared sensor, NDIR, pentahedron gas-cell

## Abstract

In order to improve the performance of the large divergence angle mid-infrared source in gas sensing, this paper aims at developing a methane (CH_4_) sensor with non-dispersive infrared (NDIR) technology using a compact pentahedron gas-cell. A paraboloid concentrator, two biconvex lenses and five planar mirrors were used to set up the pentahedron structure. The gas cell is endowed with a 170 mm optical path length with a volume of 19.8 mL. The mathematical model of the cross-section and the three-dimension spiral structure of the pentahedron gas-cell were established. The gas-cell was integrated with a mid-infrared light source and a detector as the optical part of the sensor. Concerning the electrical part, a STM32F429 was employed as a microcontroller to generate the driving signal for the IR source, and the signal from the detector was sampled by an analog-to-digital converter. A static volumetric method was employed for the experimental setup, and 20 different concentration CH_4_ samples were prepared to study the sensor’s evaluation, which revealed a 1σ detection limit of 2.96 parts-per-million (ppm) with a 43 s averaging time.

## 1. Introduction

Methane (CH_4_) is one of the simplest organic matters and is widely available in nature. It is a by-product of coal mines and can be considered as one of the most important greenhouse gases [1,2,3,4,5]. Therefore, CH_4_ detection is crucial in both industrial and in environmental scopes [3]. Numerous techniques have been successfully used in CH_4_ detection, such as tunable diode laser absorption spectroscopy (TDLAS) [6,7,8], quartz-enhanced photoacoustic spectroscopy (QEPAS) [9,10,11], and cavity enhanced absorption spectroscopy (CEAS) [12,13,14]. These kinds of CH_4_ sensors target very low detection limits (DL) at parts-per-billion (ppb) or even parts-per-trillion (ppt) levels. However, to achieve high accuracy and low DL, expensive lasers (tens of thousands of US dollars) are required, which makes these kinds of sensors unsuitable for wide industrial production or ordinary breath monitoring. The advantages of semiconductor gas sensors, specifically resistance and electrochemical types, consist of a small sizes (millimeters) and a low detection limits (ppb level); therefore, wide literature is available on such technology [15,16,17]. However, most of the available units are laboratory products and not yet commercialized, representing a boundary for the mass production and its related marketing. 

Incandescent lamp is one kind of mid-infrared source which has been demonstrated to be successfully employed in gas detectors based on infrared absorption due to its small size and low cost (a few US dollars) [18,19]. Non-dispersive infrared (NDIR) is a proper method for this kind of sensor [20,21,22]. However, due to the large divergence angle, such a kind of source cannot be condensed into the multi-pass gas cell for hundreds of reflections without mode overlap. Therefore, the design of a gas cell with small size and long optical path represents a challenge as regards the incandescent lamp within the infrared absorption sensor. In this paper, a paraboloid concentrator, two biconvex lenses and five planar mirrors were used to design the pentahedron gas-cell structure. A CH_4_ sensor was integrated with this gas cell and the achieved detection limit could reach up to sub-ppm level. 

## 2. Pentahedron Gas-Cell Structure

The selected light source was an IRL715, which is a kind of incandescent lamp widely used in NDIR sensor applications. The divergence angle of the IRL715 is ~360°. To increase the optical length and reduce the gas-cell volume, a paraboloid concentrator was employed to collect the divergent light and two biconvex lenses were placed at the in- and out-let gas cells. For getting parallel light, five planar mirrors were setup with a pentahedron gas-cell for increasing the optical path. 

### 2.1. Paraboloid Concentrator

**Figure 1:**
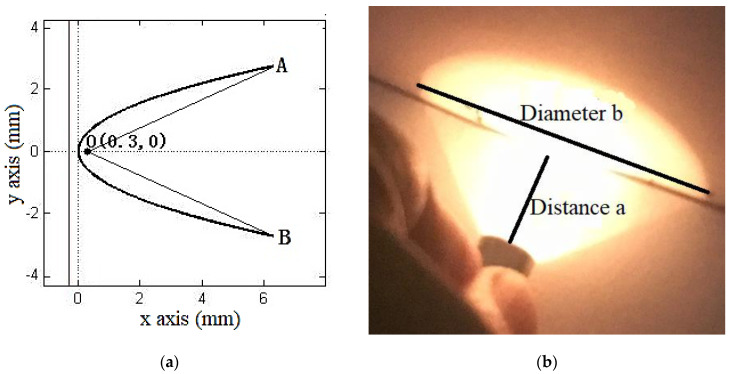
(**a**) The mathematical model and (**b**) experimental tests of paraboloid concentrator.

To focus the divergence light from the IRL715, a paraboloid concentrator was designed according to the mathematical model shown in Figure 1a. The focus of the parabola was O (0, 3, 0), the focal length was 0.66 mm, the OA and OB distances were set to 6.5 mm, and the thickness was 0.5 mm. The outlet angle can be calculated as per Equation (1):

(1) $$∠AOB=2\times arctan\left(\frac{y_{A}}{x_{A}-x_{o}}\right)=48.502^{{}^{\circ}}$$

where *y_A_* and *x_A_* are, respectively, the *y* and *x* axis coordinates of point *A*. 

The experiment was carried out to verify the design, which is shown as Figure 1b. To calculate the divergence angle, experimental tests under different diameters were carried out, the distance *a* and diameter *b* of the spots were recorded and are reported in Table 1. 

According to the triangle similarity, the divergence angle can be calculated by Equation (2):

(2) $$\alpha =2arctan(\frac{b_{2}-b_{1}}{a_{2}-a_{1}})$$

where *a* and *b* can be selected from Table 1. The calculated average divergence angle *α* was 43.414° and the error to the theoretical calculation by Equation (1) was only ~10%. 

### 2.2. Pentahedron Structure

**Figure 2:**
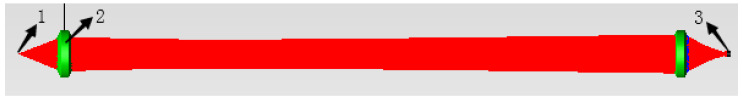
Optical simulation of the two convex lenses, where 1 is the output beam from the paraboloid concentrator, 2 is the convex lens and 3 is the detector.

To parallel the beam, a convex lens was employed at the outlet of the output beam from paraboloid concentrator, and to converge the beam, another convex lens was used before the detector. The optical simulation is shown in Figure 2. 

**Figure 3:**
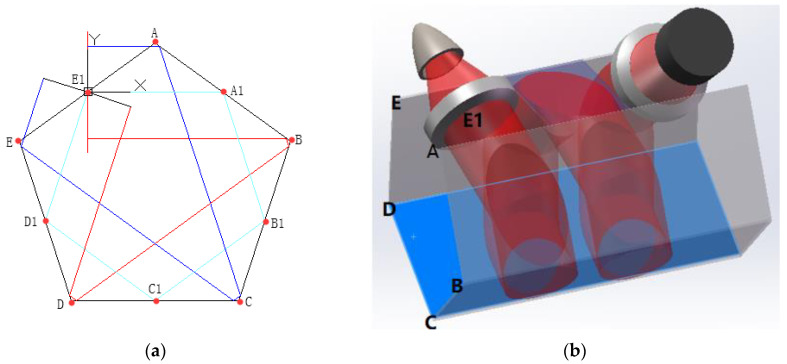
(**a**) Mathematical model of cross-section and (**b**) three-dimensional spiral structure of the pentahedron gas-cell.

With the aim of reducing the size, shown in Figure 2, five planar mirrors were added in order to build a small-size gas cell. The cross-section mathematical model is shown in Figure 3a and the three-dimensional spiral structure is shown in Figure 3b. E1 is the center of both convex lenses—the distances between the lens to the source and the lens to the detector are both 12 mm. AB, BC, CD and DE are planar mirrors with diameters of ~9.7 mm, which is consistent with the diameter of the parallel beam. 

In order to avoid overlapping issues in the spiral optical path, the lenses should be placed at a tilt angle *β*, which can be calculated by Equation (3):

(3) $$\beta \geq arctan\left(\frac{D_{\mathrm{path}}}{D_{\mathrm{beam}}}\right)$$

where $D_{\mathrm{path}}$ is the optical path under one plane and $D_{\mathrm{beam}}$ is the diameter of the beam. According to Equation (3), *β* was chosen to be 10.2°, and the fifth mirror was placed between two convex lenses for increasing the optical path. According to the mathematical model implementation, the optical path from the source to the detector can reach 170 mm with a volume of 19.8 mL. 

## 3. NDIR Sensor Configuration 

The NDIR CH_4_ sensor consists of optical parts and electrical parts. The core of optical part is the pentahedron gas-cell—the electrical part contains the hardware connection and software program. 

### 3.1. Sensor Architecture

**Figure 4:**
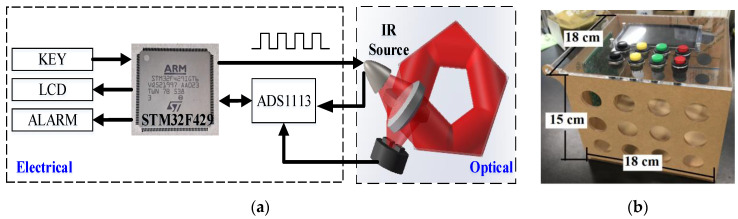
(**a**) Schematic diagram of the NDIR CH_4_ sensor, including an electrical and an optical part and (**b**) photo of the sensor.

The schematic diagram of the NDIR CH_4_ sensor is depicted in Figure 4a, including electrical and optical part. A photo is shown in Figure 4b with the dimensions of 18 (L)×18 (W)×15(H) cm. The pentahedron gas cell was integrated in the optical part of the sensor. In the electrical part, a STM32F429 (STMicroelectronics, Geneva, Switzerland) was employed as the MCU (Microcontroller Unit) of the whole system, which generated a 4 Hz square-wave to drive the IR source (IRL715, Perkin Elmer) and collected output signals from the detector (PYS3228TCG5.2, Excelitas, USA) to the analog-to-digital converter (ADC, ADS1113, Texas Instruments, USA). 

### 3.2. Hardware Design

**Figure 5:**
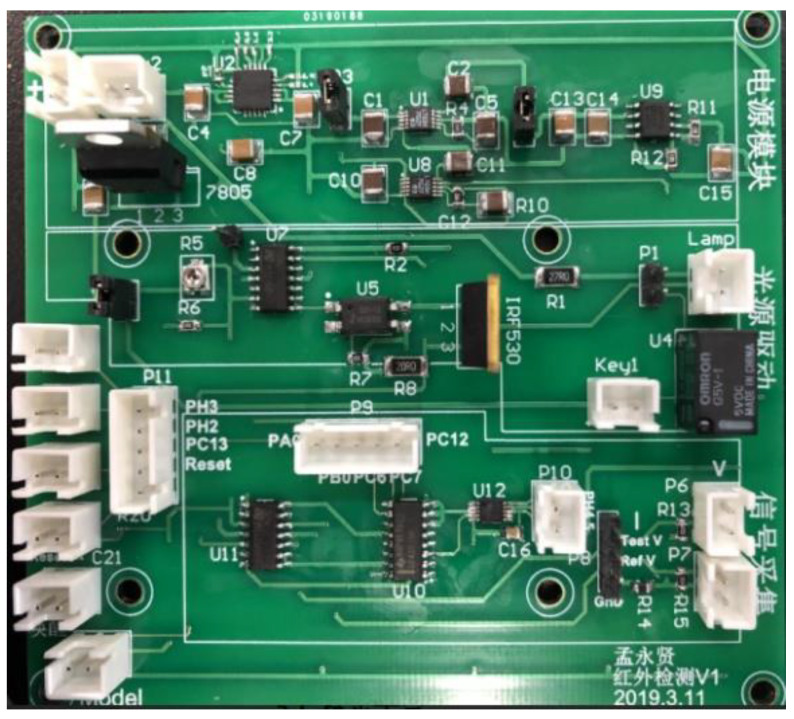
Photo of circuit board.

**Figure 6:**
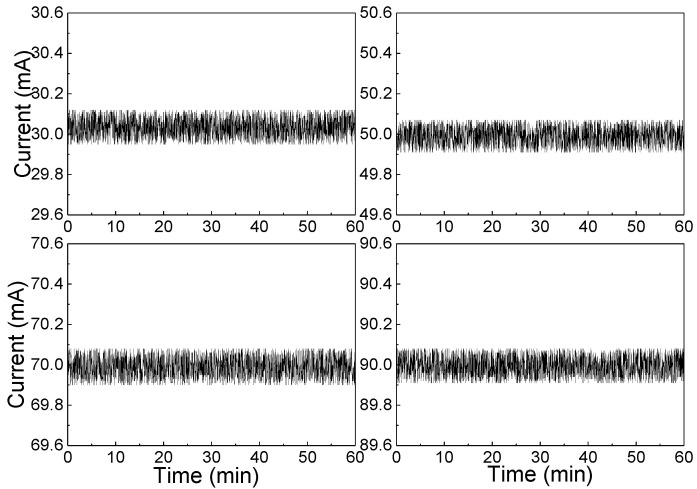
Flow chart of main detection procedure of STM32 processor.

To get the stable optical power, a constant current driver circuit was designed with a MOSFET as a switch and an amplifier as a feedback controller—the circuit board is shown in Figure 5. This board can generate a constant current for IRL715 with a power of 0.5 W. For testing the stability of this circuit, four currents of 30 mA, 50mA, 70 mA and 90 mA were set and tested for 1 h. From Figure 6, the fluctuation of the current is ±0.083 mA.

**Figure 7:**
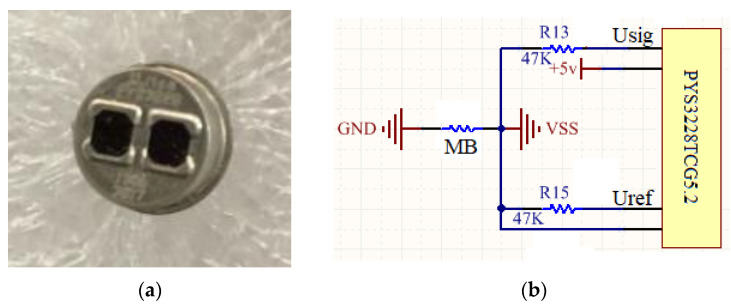
(**a**) The mathematical model and (**b**) experimental tests of paraboloid concentrator.

The detector is shown in Figure 7a, which is a pyroelectric sensor with two channels of 3.31 um and 4.0 um. The signal output (U_sig_) from the channel with 3.3 um optical filter was absorbed by CH_4_, which can be used for CH_4_ concentration detection. The one from the channel with 4.0 um optical filter window (U_ref_) without any absorption, which can be used as the reference channel for noise suppression. There are four pins on the detector, which were connected to the power, ground and the U_sig_ and U_re_. The electrical connection is shown in Figure 7b—two 47 K resistances were employed for impedance matching and a magnetic bead (MB) was selected for suppressing the electrical influence from power supply.

In addition, the concentration threshold can be set through a keyboard. The real-time concentration can be shown on an LCD and stored in a secure digital (SD) card. Once the detected CH_4_ concentration result is higher than the set threshold value, an acousto-optic alarm is be triggered.

### 3.3. Signal Acquisition and Processing

**Figure 8:**
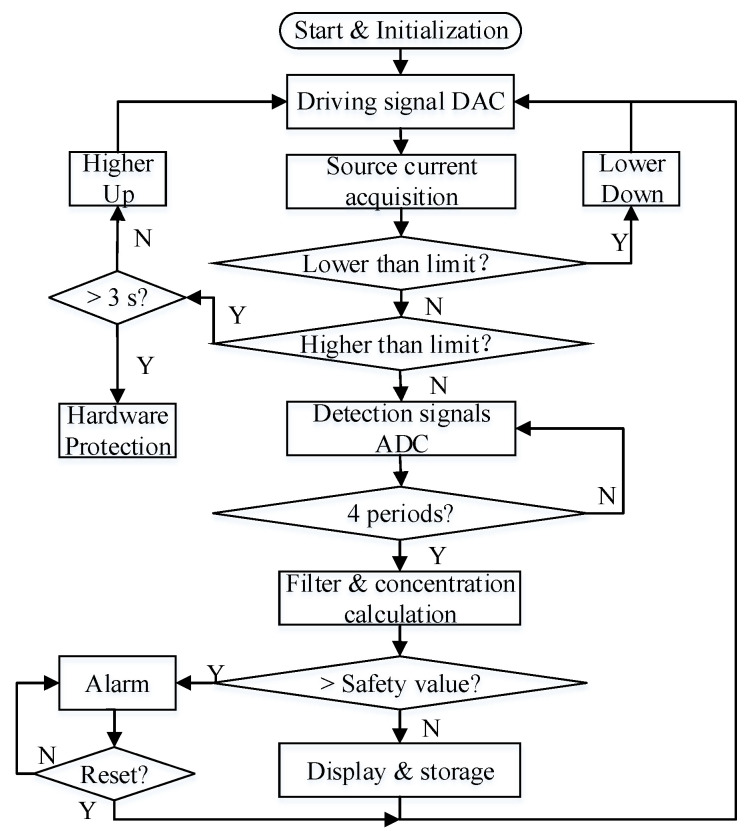
Flow chart of the sensor’s signal acquisition and processing.

The flow chart of the sensor’s signal acquisition and processing is depicted in Figure 8. In order to get a stable signal from the sensor, a constant current to the IR source is a primary key. A negative feedback programmable constant current was developed for this sensor. The ADS1113 sampled the real-time current signal from the IR source in a continuous conversion mode at the sampling rate of 475 samples per second (SPS). The sampled current was compared with the preset maximum–minimum current bounds and adjusted in real-time. If the current value was higher than the upper limit for more than 3 s, the hardware protection circuit will be triggered. 

The analog signal from the detector was converted by the ADS1113 to a digital signal and sent to the MCU. A program of mean filter was compiled to suppress the noise. The concentration was calculated and compared to the threshold, once the value exceeded the safety value, the acousto-optic alarm was triggered and set to wait for reset. If the concentration was below the threshold, it would be displayed on the LCD and stored in the SD card. 

## 4. Experimental Tests and Results

### 4.1. Expeirmental Setup

**Figure 9:**
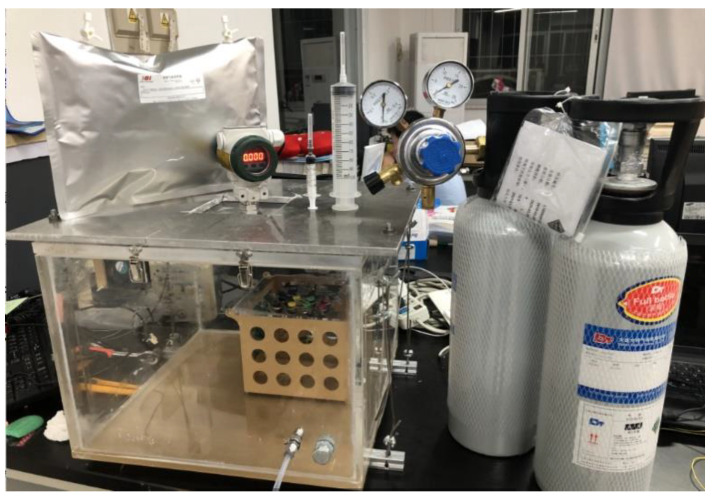
Photo of the air-tight chamber.

An air-tight chamber made of acrylic plate was designed with a size of 40 cm × 40 cm × 30 cm and a volume of 48 liters, as shown in Figure 9. A 1/4 inch (6.35 mm) three-way ferrule ball valve was connected with the M12 × 1.25 thread to ensure the air tightness. The valve had two inlets—one was connected with a pure N_2_ cylinder and the other was connected with a 99.999 % CH_4_ cylinder. Based on ISO 6144: 2003 [23], the concentration in the chamber can be calculated as per Equation (4):

(4) $$\phi \left(x\right)=\frac{p_{1}\times V_{xg}}{p_{2}\times V_{cg}+p_{1}\times V_{xg}}$$

where ϕ(x) is the target concentration of CH_4_, $p_{1}$ and $p_{2}$ are the pressure values inside the chamber, respectively, before and after the distribution, $V_{cg}$ is the volume of the chamber and $V_{xg}$ is the volume of the injected gas. According to Equation (4), 20 different concentrations (*C*) with volumes (*V*) for calibration were calculated and are listed in Table 2. 

### 4.2. Sensor Evaluation

**Figure 10:**
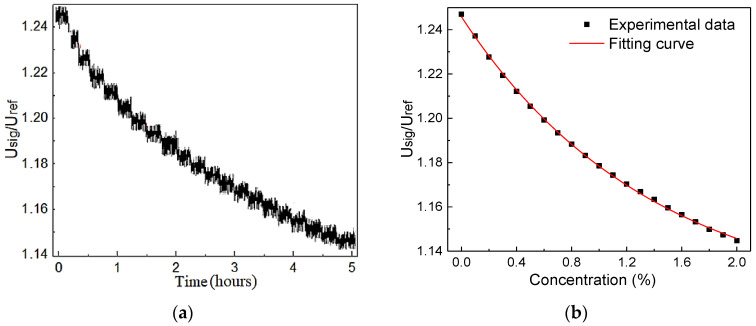
(**a**) The measured voltage ratio between the two channels from the detector versus calibration time *t* for 20 CH_4_ concentration levels. (**b**) Experimental data and fitting curve.

The sensor calibration was carried out by using 20 different CH_4_ concentration samples from Table 2, and the results are shown in Figure 10a. The voltage ratio between the two channels from the detector, signal (*U*_sig_) and reference (*U*_ref_), were used for noise suppression. The averaged values and fitting curve are shown in Figure 10b. The fitting curve indicates a good exponential relationship (R-square value: 99.79 %), which is consistent with the Lambert–Beer’s Rule [24], expressed by Equation (5):

(5) $$\frac{U_{\mathrm{sig}}}{U_{\mathrm{ref}}}=1.11515+0.13069{exp}^{\left(\frac{-C}{1.37711}\right)}$$

**Figure 11:**
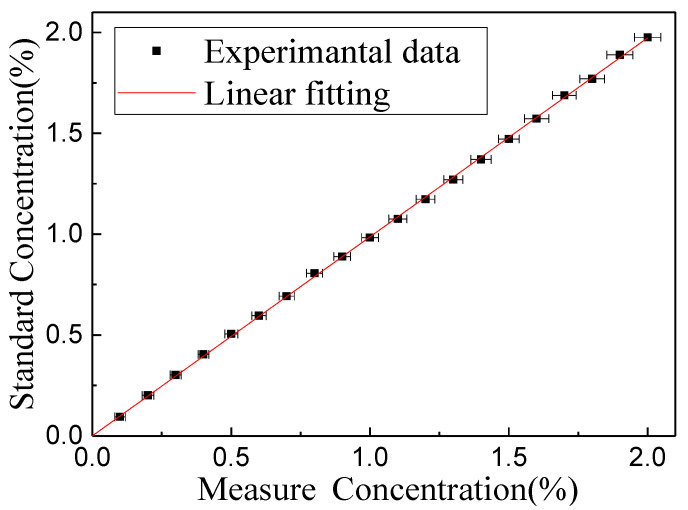
Fitting curve of the measured and standard concentration.

For evaluating the accuracy of the CH_4_ sensor, the error bar was employed to compare the measured and the standard concentration, as shown in Figure 11, where the fitting curve indicated a good linear relationship (R-square value: 99.95 %). 

**Figure 12:**
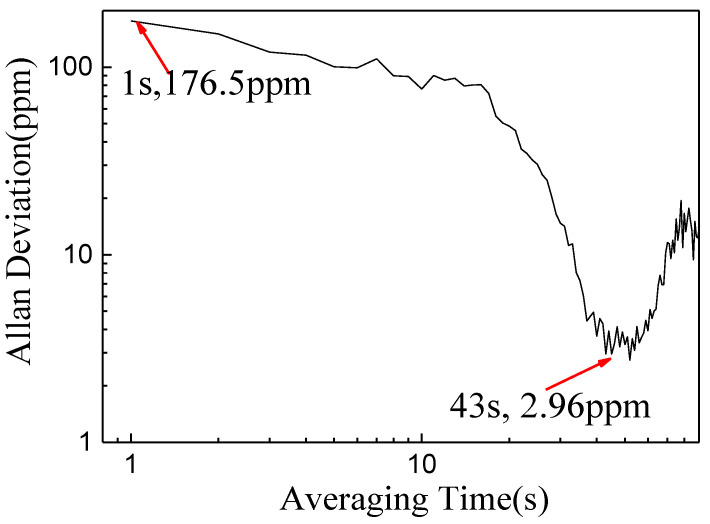
Allan variation plot as a function of averaging time.

Measurement of a CH_4_ sample with 2 % concentration over a period of ~1 hour was performed. Figure 12 shows the Allan variation, which is defined as Equation (6), which calculates one half of the averaging time of the squares of the differences between successive readings of the frequency deviation sampled over the sampling period [25]. The Allan variation is ~176.5 parts-per-million (ppm) with a 1 s averaging time and an optimum averaging time of 43 s, corresponding to a detection limit of 2.96 ppm.

(6) $$\sigma_{y}^{2}\left(\tau \right)=\frac{1}{2\tau^{2}}\left[{\left(x_{n+2}-2x_{n+1}+x_{n}\right)}^{2}\right]$$

where *τ* is the averaging time, $x_{n}$ is the measurement result at time *t*. 

## 5. Conclusions

A portable CH_4_ sensor using a compact pentahedron gas-cell, based on NDIR technology, was demonstrated. The gas-cell with a paraboloid concentrator, two biconvex lenses and five planar mirrors was realized with a 170 mm optical path length and a 19.8-mL volume. Statistic distribution was used in the CH_4_ measurement for evaluating the sensor’s performance and 20 samples with different concentration levels were prepared for calibration. An Allan variation analysis yielded a detection sensitivity of 2.96 ppm with a 43 s averaging time.

## Figures and Tables

**Table 1:** Light spot divergence angle test records.

Distance *a* (mm)	Diameter *b* (mm)
5	21.5
10	31
20	49.5
30	68.5
40	87.1
60	12.65

**Table 2:** The calculated CH_4_ concentration values and volumes for calibration.

C (%)	V (mL)	C (%)	V (mL)	C (%)	V (mL)	C (%)	V (mL)	C (%)	V (mL)
0.1	48.1	0.2	96.2	0.3	144.4	0.4	192.8	0.5	241.7
0.6	289.7	0.7	339.7	0.8	387.1	0.9	435.9	1.0	484.8
1.1	533.9	1.2	583.0	1.3	632.2	1.4	681.5	1.5	731.0
1.6	780.5	1.7	830.1	1.8	880.0	1.9	929.7	2.0	979.6
